# Avian Surveys in the Korean Inner Border Area, Gimpo, Republic of Korea

**DOI:** 10.3897/BDJ.8.e56219

**Published:** 2020-11-06

**Authors:** Hyun-Ah Choi, Bernhard Seliger, Nial Moores, Amaël Borzée, Chong Hwi Kevin Yoon

**Affiliations:** 1 Hanns Seidel Foundation Korea, Seoul, South Korea Hanns Seidel Foundation Korea Seoul South Korea; 2 OJeong Eco-Resilience Institute, Korea University, Seoul, South Korea OJeong Eco-Resilience Institute, Korea University Seoul South Korea; 3 Birds Korea, Busan, South Korea Birds Korea Busan South Korea; 4 Laboratory of Animal Behaviour and Conservation, College of Biology and the Environment, Nanjing Forestry University, Nanjing, China Laboratory of Animal Behaviour and Conservation, College of Biology and the Environment, Nanjing Forestry University Nanjing China

**Keywords:** border area, waterbirds, bird conservation, Neutral Zone, wetlands, conservation, Korean peninsula

## Abstract

**Background:**

Birds are useful environmental indicators as their presence reflects the health of the food web. Bird occurrence, rarity and abundance are reliable indicators of ecosystem health. Monitoring of avian populations in the Republic of Korea (ROK) is a primary requirement due to plummeting populations and the risks to threatened species. The Ministry of Environment of ROK started conducting winter bird censuses in 1999, including inland areas and coast areas, such as Cheorwon, Yeoncheon, Junam Reservoir and Han River. Cheolwon, Yeoncheon and some extent islands in the West Sea have been survey extensively due to iconic bird species, such as White-naped Crane (*Grus vipio*) or Red-Crowned Crane (*Grus japonensis*) wintering there. However, the winter bird census has not covered Yu Islet, Han River Estuary. Yu Islet is located within the Han River Estuary, a protected wetland in the Neutral Zone between the two Koreas and north of Gimpo in the ROK. The Islet currently supports a large, mixed breeding colony of waterbirds, such as one of the nation’s largest concentration of breeding Great Cormorants (*Phalacrocorax carbo*) and smaller numbers of breeding Black-faced Spoonbill (*Platalea minor*), Grey Heron (*Ardea cinereal*), Great Egret (*Ardea alba*) and Intermediate Egret (*Ardea intermedia*). Access to the area has long been restricted for military reasons, but recently, regular survey activity is possible supported by Gimpo City and the military base in Gimpo from November 2018.

**New information:**

Here, we provide data demonstrating that Yu Islet is important for breeding for waterbirds; and that the northern Gimpo part of Han River Estuary is also internationally important for waterbirds during the migratory bird season, as defined by the Ramsar Convention (Ramsar 1971, RRC-EA 2017). In particular, four waterbird species were found during the survey in the Main Survey Area: Swan Goose (*Anser cygnoides*), Taiga Bean Goose (*Anser fabalis*), Tundra Bean Goose (*Anser serrirostris)* and Greater White-fronted Goose (*Anser frontalis*). Once considered widespread in East Asia and abundant, the world population of Swan Goose is now estimated at only 60,000 - 78,000 individuals (Wetlands International 2020) and the species is assessed by BirdLife International as globally Vulnerable (BirdLife International 2020). The 1,010 Swan Goose (*Anser cygnoides*) counted on the vegetated mudflats at Jogang-Ri in the Main Survey Area on 27 November 2018 represents more than 1% of the total world population of this species. Notably, it is also the highest count of this species in the ROK for at least a decade. The count confirms the continuing international importance of the Han River Estuary for the survival of the Swan Goose. The number counted in November had fallen to 250 by 28 December 2018; and none was recorded in the Main Survey Area in January or February 2019. Although searched for in March, none was noted during the northward migration either. The surveys also found small numbers of nationally-scarce Grey-capped Pygmy Woodpecker (*Yungipicus canicapillus)* in several areas of woodland surrounded by the Han River Estuary. By selecting the most species-rich count within a given month in each of the two survey sectors, the number of species we recorded ranged from a minimum 29 in January to a maximum of 65 in April 2019. Based on the species recorded, the survey area is clearly important for avian conservation. Its importance derives from the combination of the extensive areas of high-quality wetland and its geographic location within one of the Korea Peninsula’s largest and most important remaining wetland ecosystems, the Han River Estuary. Our surveys resulted in the detection of a substantial number of bird species, especially in March and April when forest-breeding birds are more obviously vocal. The survey result is provided in the supplementary material (Suppl. material 1).

## Introduction

Bird monitoring in the Republic of Korea (ROK) is important because of the current decline in population sizes affecting all species (Lee and Miller-Rushing 2014). As a result, timely conservation actions are required to avoid species extinctions and current society dynamics seem to indicate citizen support for such projects (Borzée et al. 2019). The need for conservation activities and habitat protection is especially true in coastal areas as development projects on riverine habitats and tidal flats have strongly impacted the quality of the habitat and make resources required by birds difficult to acquire (Lee et al. 2015, Lee et al. 2017). Yu Islet is within the Neutral Zone and it is difficult to enter. Entrance requires permission from the authorities of the two Koreas and the United Nations Command Military Armistice Commission (Anonymous 2017). However, the surrounding area of Yu Islet, Wolgot-Myeon, is managed by Gimpo City, ROK. The area is important to nesting waterbirds, including the globally Endangered Black-faced Spoonbill (*Platalea minor; Kang et al. 2016*), based on surveys conducted from the mainland. Waterbird surveys in other sections of the Han River Estuary in the Republic of Korea part have also already identified these areas as internationally important following the definition of the Ramsar Convention (MOE 1999-2017, EAAFP 2019).

## General description

### Purpose

**Figure 1.**
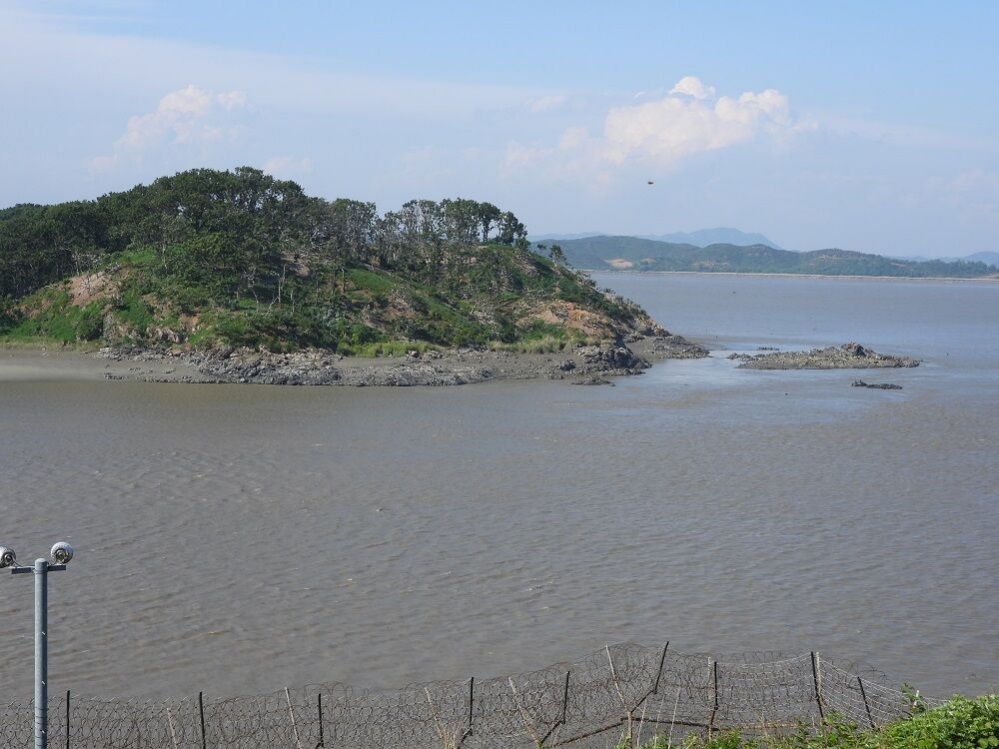
Yu Islet in the Han River Estuary – hundreds of Great Cormorants, Great Egrets, Grey Herons and also some Blackfaced Spoonbills breed here. In the background is the coastline of South Hwanghae Province, DPRK (Photo by Bernhard Seliger on 27 May 2020)

**Figure 2.**
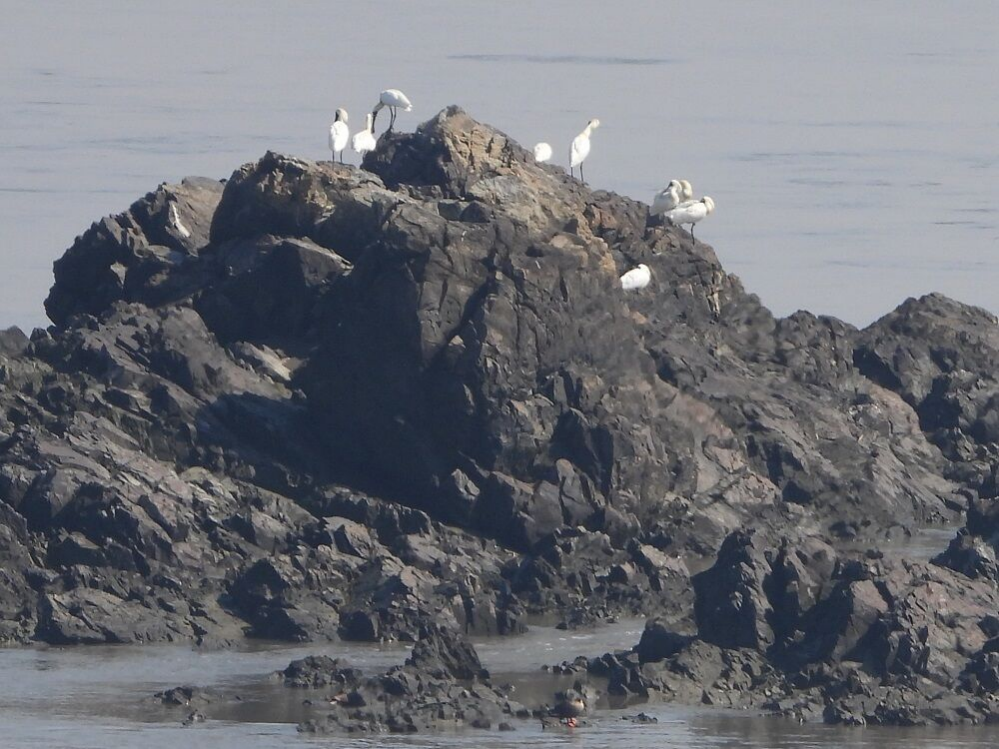
Black-faced Spoonbill *Platalea minor* at Yu Islet (Photo by Bernhard Seliger on 24 March 2020)

The aim of this work was to conduct an inventory of the waterbirds in the inner border area of Gimpo in the ROK. In addition, the present survey aims to determine whether Yu Islet is still important for nesting waterbirds; and whether the northern Gimpo part of the Han River Estuary is also internationally important for waterbirds, as defined by the Ramsar Convention to help provide information for future land use plans (Figs 1, 2).

## Project description

### Title

Ecological survey of Yu Islet and adjacent habitats in northern Gimpo

### Personnel

The survey was conducted during seven months between November 2018 and April 2019 by experienced birdwatchers: Nial Moores, Bernhard Seliger and Hyun-Ah Choi.

### Study area description

**Figure 3.**
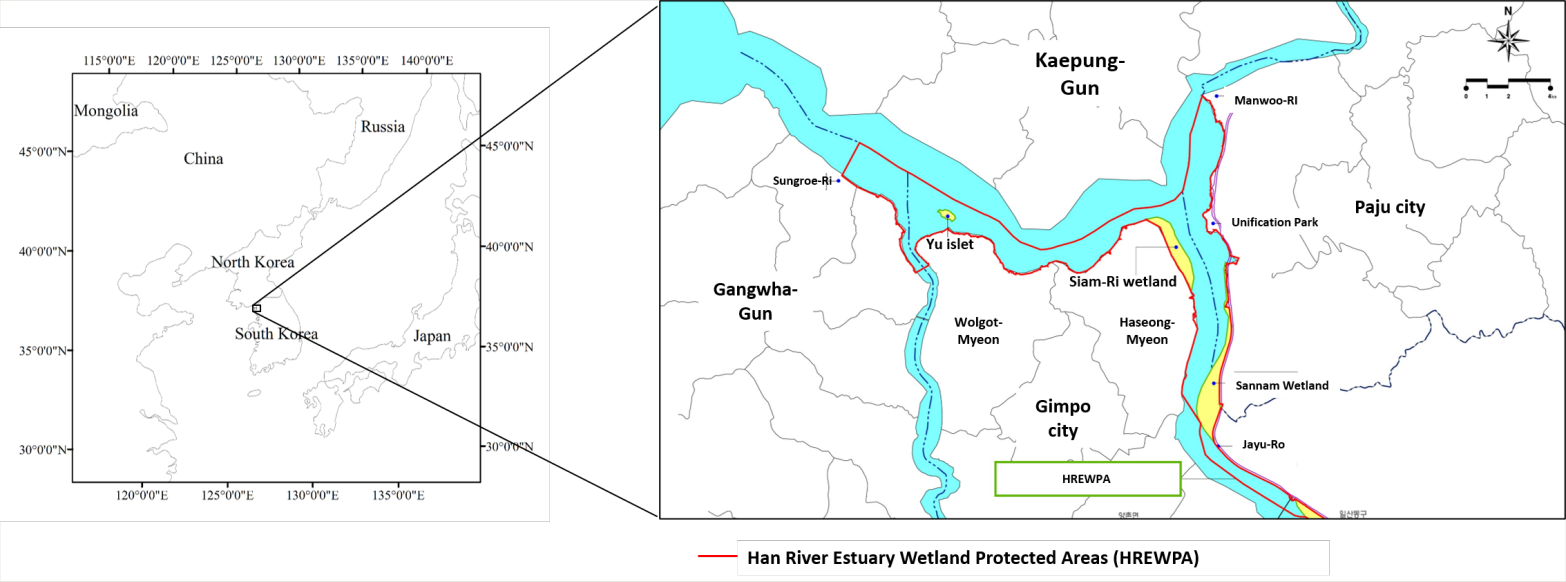
Han River Estuary Wetland Protected Area, including the survey area (Sourced by our own compilation, based on the map of Han River Estuary Wetland Protected Area by MOE)

**Figure 4.**
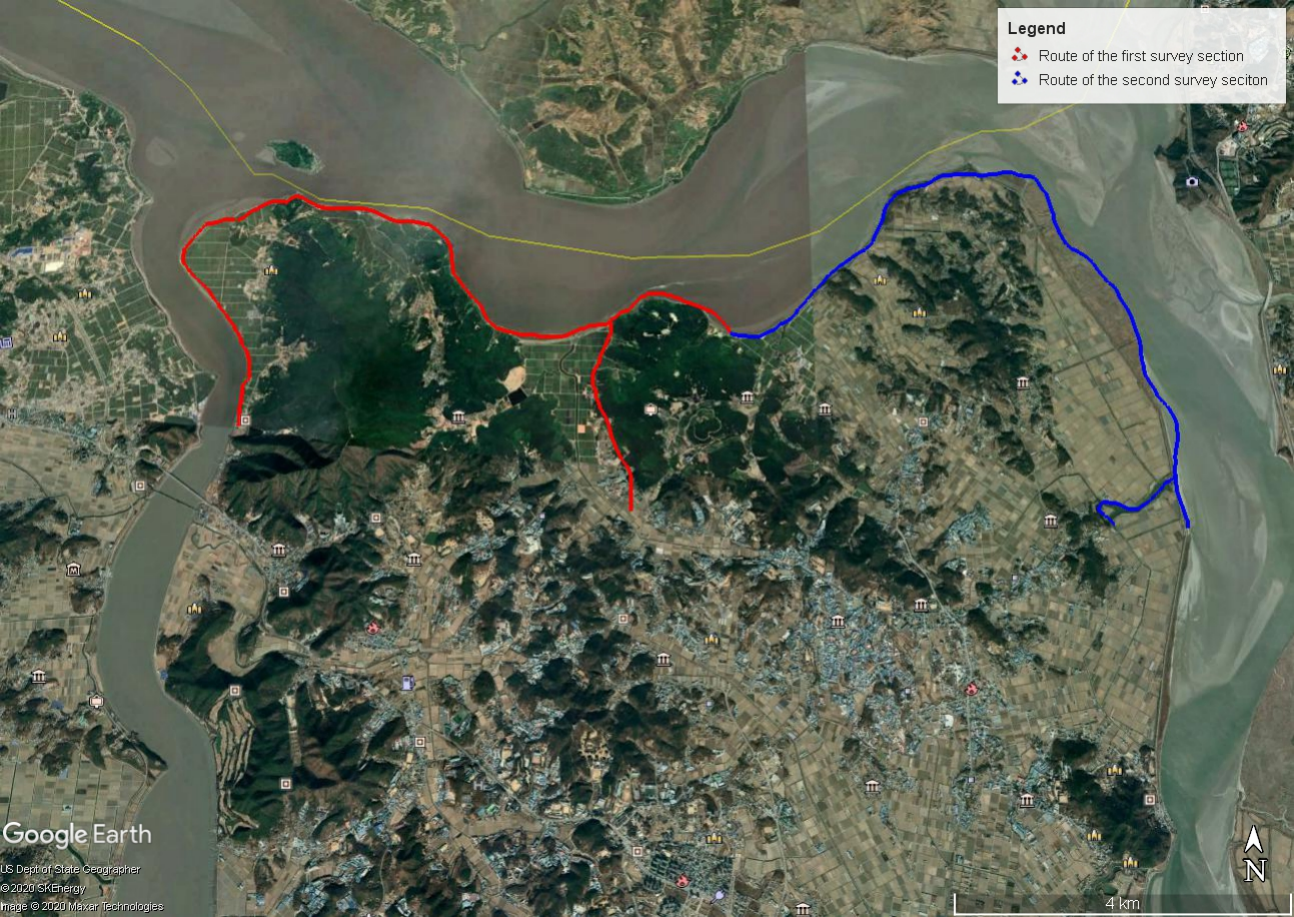
The route of the survey (Sourced by our own compilation, based on Google Earth). Red indicates the Main Survey Area, blue indicates the Supplementary Survey Area.

The study area is part of Han River Estuary Wetland Protected Area designated on 17 April 2006 by the Ministry of Environment (MOE), ROK (Fig. 3, Fig. 4). The total area is 60.668 km^2^. This area is also a Military Installation Protected Area.

## Sampling methods

### Study extent

This study covers Yu Islet and surrounding area.

### Sampling description

The survey was conducted 11 times from 09:30 to 17:00 (Korea Standard Time) between 27 November 2018 and 26 April 2019 by experienced birdwatchers.

We divided the survey into two sections and used fixed survey points, determined in advance, as well as opportunistic survey points. Given the disturbance in the area, as well as the feeding habits of many migratory birds, like geese, a completely fixed system of survey points would not have made sense. Furthermore, surveying was partly restricted or hindered by military escort, though not too severely. The survey team used a car (sometimes followed by another military vehicle) to survey the area, with points to leave the car and conduct scans. The first survey section of the area included Yu Islet (as visible from the shore) and the adjacent habitats southwards, all within 5 km of the Islet itself. This area was referred to as the Main Survey Area. The second survey section, referred to as the Supplementary Survey Area, included many of the superficially similar habitats and immediately adjacent habitats in the northeast of Gimpo, from the forested ridge bordering the Main Survey Area to the east of Aegibong, east to Siam-Ri and south to the north of Seoktan-Ri, at most up to 10 km southeast of Yu Islet. It also included birds on the DPRK side of the river. We visited the survey area at least two times per month between January and April in 2019.

We focused the counting efforts on bird species found in open wetland habitats, as defined by the Ramsar Convention (i.e. in rice-fields; along streams and waterways; the river-edge; and the main river). We visited the survey sections in sequence. However, during the surveys, we also counted every individual bird that we either heard or saw from 1) a slowly moving vehicle (average < 20 km/h); 2) from points in areas with many birds. The survey team was equipped with a Swarovski Scope, sometimes a second Swavorski Scope, as well as binoculars. Counts were accurate for smaller groups of birds, but had to be estimated for larger groups of birds, sometimes up to 5,000 birds in a single flock. Double checks by experienced birdwatchers in the team, wherever possible, however, confirmed the size of the flocks with a high degree of confidence. The survey team, for this reason, worked with birdwatchers with decades of experience on the Korean Peninsula.

## Geographic coverage

### Description

Yu Islet, Wolgot-Myeon and Siam-Ri wetland, Haseong-Myeon, Gimpo City of ROK, 126°32'-126°39'E and 37°45-37°46'N.

## Temporal coverage

**Data range:** 2018-11-27 – 2019-4-26.

## Usage licence

### Usage licence

Creative Commons Public Domain Waiver (CC-Zero)

## Data resources

### Data package title

Avian Survey result

### Number of data sets

1

### Data set 1.

#### Data set name

Avian Survey result

#### Data format

CSV

#### Number of columns

21

#### Data format version

Darwin Core Archive

#### 

**Data set 1.** 

Column label	Column description
basisOfRecord	The specific nature of the data record.
recordedBy	A list of names of people, groups or organisations responsible for recording the original Occurrence.
eventDate	The date-time or interval during which an Event occurred.
eventTime	The time or interval during which an Event occurred.
eventRemarks	Weather conditions.
Continent	The name of the continent in which the Location occurs.
countryCode	The standard code for the country in which the Location occurs.
country	The name of the country or major administrative unit in which the Location occurs.
locality	The specific description of the place.
locationRemarks	Comments or notes about the Location.
decimalLatitude	The geographic latitude (in decimal degrees, using the spatial reference system given in geodeticDatum) of the geographic centre of a Location.
decimalLongitude	The geographic longitude (in decimal degrees, using the spatial reference system given in geodeticDatum) of the geographic centre of a Location.
geodeticDatum	The ellipsoid, geodetic datum or spatial reference system (SRS) upon which the geographic coordinates given in decimalLatitude and decimalLongitude are based.
coordinateUncertaintyInMetres	The horizontal distance (in metres) from the given decimalLatitude and decimalLongitude describing the smallest circle containing the whole of the Location. Leave the value empty if the uncertainty is unknown, cannot be estimated or is not applicable (because there are no coordinates). Zero is not a valid value for this term.
class	Class name
vernacularName	A common or vernacular name.
scientificName	An identifier for the nomenclatural (not taxonomic) details of a scientific name.
individualCount	The number of individuals represented present.
identificationReferences	List of references (publication) used in the Identification.
licence	A legal document giving official permission to do something with the resource.
occurrenceID	An identifier for the Occurrence (as opposed to a particular digital record of the occurrence).

## Supplementary Material

43E6ADB3-AEFB-5614-9A18-389A7FA27C83 10.3897/BDJ.8.e56219.suppl1 Supplementary material 1 Avian Survey result Data type Occurrences Brief description The survey area dataset provides the list of avian survey results along with all the relevant information available. File: oo_428625.csv https://binary.pensoft.net/file/428625 Hyun-Ah Choi and Chong Hwi Kevin Yoon
